# Supplementary material for: The Internet, Apps, and the Anesthesiologist
Source: Healthcare (Basel). 2023 Nov 20;11(22):3000. doi: 10.3390/healthcare11223000 (PMC10671284; doi:10.3390/healthcare11223000)
Supplement: Supplementary file 1 [file healthcare-11-03000-s001.zip › healthcare-2698154-supplementary.pdf]

Supplementary Materials

Table S1: Examples of Apps used in Anesthesia

App name	App type	Clinical Area	Description	Cost	URL
**General clinical support**					
5 Minute Anesthesia Consult	Healthcare provider decision support<br>Drug references, medication management<br>Reference texts/journals, training and education<br>Patient centred app	Pre-operative setting<br>General anesthesia<br>Surgical specialty	Thorough pathology-based app extending from pre-operative management to intra-operative management	Free<br><br>In-app purchase $119.99	https://apps.apple.com/us/app/5-minute-anesthesia-consult/id1102384412
AAGBI (Association of Anaesthetists of Great Britain and Ireland)	Healthcare provider decision support<br>Drug references, medication management<br>Reference texts/ journals, training and education<br>Medical calculators<br>Healthcare provider communication/ referral coordination	Pre-operative setting<br>General anesthesia<br>Surgical specialty<br>Regional anesthesia	Centralised hub for interaction between anaesthetists, guidelines, and education	Free	https://apps.apple.com/us/app/association-of-anaesthetists/id913194458
Anesthesia 411	Healthcare provider decision support<br>Drug references, medication management<br>Reference texts/journals, training and education	General anesthesia<br>Surgical specialty	Specific procedures and the integral information for anaesthetists to perform them safely	$3.99	https://apps.apple.com/us/app/anesthesia-411/id449473295
Anesthesia Central	Healthcare provider decision support<br>Drug references, medication management<br>Reference texts/ journals, training and education	General anesthesia<br>Surgical specialty	Combination resource allowing for multiple uses across several domains	Free<br><br>In-app purchases $99.99 - $169.99	https://apps.apple.com/us/app/anesthesia-central/id347656772

| App name | App type | Clinical Area | Description | Cost | URL |

ASRA Coags	Healthcare provider decision support<br>Drug references, medication management<br><br>Patient centred app	Pre-operative<br>Regional anesthesia<br>Pain medicine	Guidelines provided for regional anesthesia and pain management	$3.99	https://apps.apple.com/us/app/asra-coags/id858796572
Anesthesia Pre-Op	Healthcare provider decision support<br>Patient centred app	Pre-operative setting	Provides checklist for pre-operative patient assessment	Free	https://play.google.com/store/apps/details?id=com.mobincube.anesthesia_pre_op.sc_HNMR4H
MyATLS	Healthcare provider decision support<br>Drug references, medication management<br><br>Medical calculators<br>Reference texts/journals, training and education	General anesthesia<br>Surgical specialty	Easy to access trauma based clinical decision support tool	$1.22 - $9.77 per item	https://play.google.com/store/apps/details?id=com.echo.myatls
Regional Anesthesia Reference	Drug references, medication management<br>Medical calculators<br>Reference texts/journals, training and education	Regional anesthesia	Provides education about regional anesthesia, recommended analgesia and calculator	Free	https://play.google.com/store/apps/details?id=com.awalters. regionalanestheiaatlas
TTE Standard Views	Reference texts/journals, training and education	Pre-operative	Provides 3-dimensional context for images viewed in transthoracic echocardiography	$4.99	https://apps.apple.com/us/app/tte-standard-views/id660856485
ECHO Views - Transesophageal Echocardiography	Reference texts/journals, training and education	Pre-operative	Transesophageal Echocardiography educational resource	$9.99	https://apps.apple.com/us/app/echo-views-transesophageal-echocardiography/id447218400

App name	App type	Clinical Area	Description	Cost	URL
iTIVA Anesthesia	Reference texts/journals, training and education	General anesthesia	Simulation based learning of TIVA	Free<br>In-app purchase<br>$9.99	https://apps.apple.com/us/app/itiva-anesthesia/id635652167
Medscape	Healthcare provider decision support<br>Reference texts/ journals, training and education	Pre-operative<br>General anesthesia<br>Paediatric setting<br>Surgical specialty<br>Regional anesthesia; Pain	Search engine database	Free	https://play.google.com/store/apps/details?id=com.medscape.android
**Drug References/ Medication Management**					
Anaesthetic Drugs	Drug references, medication management	General anesthesia<br>Pain medicine	Provides doses for common medications used in anesthesia	Free	https://play.google.com/store/apps/details?id=com.mobincube.anesthetic_drugs.sc_HC5XV7
Anesthesia Drugs Fast	Drug references, medication management	General anesthesia<br>Paediatric setting<br>Pain medicine	Provides doses, ranges, calculations for a wide variety of anaesthetic medications	$1.35	https://play.google.com/store/apps/details?id=com.hofficer. anesthesiadrugsfast
Epocrates	Healthcare provider decision support<br>Drug references, medication management<br>Medical calculators	General anesthesia<br>Paediatric setting<br>Pain medicine	Provides easy access to drug doses, monographs, drug calculators	Free<br>In-app purchases<br>$24.99 -<br>$349.99	https://play.google.com/store/apps/details?id=com.epocrates&hl=en_AU
**Medical Calculators**					
Calculate by QxMD	Healthcare provider decision support<br>Drug references, medication management<br>Medical Calculator	Pre-operative<br>General anesthesia<br>Surgical specialty	Centralised location for clinical point of care tools	Free	https://apps.apple.com/us/app/calculate-by-qxmd/id361811483
App name	App type	Clinical Area	Description	Cost	URL

App name	App type	Clinical Area	Description	Cost	URL
Opioid Calculator	Drug references, medication management	Pain medicine	Calculates oral morphine equivalents	Free	https://apps.apple.com/ca/app/opioid-calculator/id1039219870
Pedi-Anesth	Healthcare provider decision support<br>Drug references, medication management<br>Medical calculators	Paediatric setting	Combination resource providing multiple aids	Free	https://apps.apple.com/us/app/pedi-anesth/id1019040157
**Reference Texts**					
Atlas of Ultrasound Anesthesia	Reference texts/journals, training and education	Regional anesthesia	In depth teaching of ultrasound guided regional anesthesia techniques	Free<br>In-app purchases<br>$64.99 - $129.99	https://play.google.com/store/apps/details?id=com. mobisystems. msdict.embedded.wireless.elsevier.augra
Anaesthesiology - Paediatric & Clinical Anaesthesiology	Reference texts/journals	General anesthesia<br>Paediatric setting<br>Surgical specialty<br>Regional anesthesia<br>Pain medicine	Textbook	Free	https://play.google.com/store/apps/details?id=com. andromo.dev658544.app945759
BLOCKJOCKS Regional Anesthesia	Reference texts/journals, training and education	Regional anesthesia	Regional anesthesia educational resource	Free<br>In-app purchase<br>$19.99 - $169.99.	https://apps.apple.com/us/app/blockjocks/id1232060664
Block GuRU Lite	Reference texts/journals, training and education	Regional anesthesia	Regional anesthesia educational resource	$9.99	https://apps.apple.com/us/app/block-guru-lite/id829739487

Case Files Anaesthesiology	Reference texts/journals, training and education	General anesthesia<br>Surgical specialty<br>Regional anesthesia<br>Pain medicine	Case-based learning, with exam-based questions	Free<br><br>In-app purchase $29.99	https://apps.apple.com/us/app/case-files-anesthesiology-1e/id461592758
Echocardiography pocketcards	Reference texts/journals, training and education	Pre-operative	Flashcard based education	$3.99	https://apps.apple.com/us/app/echocardiography-pocketcards/id359676863
Read by QxMD	Reference texts/ journals, training and education	Multiple	Access to up-to-date and new journal articles, can be filtered to fit specific interests	Free	https://apps.apple.com/us/app/read-by-qxmd/id574041839
Handbook of Clinical Anesthesia	Reference texts/journals, training and education<br>Drug references, medication management<br>Medical calculators	Pre-operative<br>General anesthesia<br>Surgical specialty<br>Regional anesthesia	Provides comprehensive guidelines, recommendations, information for practicing anaesthetists	Free<br><br>In-app purchases $52.99 - 104.99	https://play.google.com/store/apps/details?id=com.medpresso. Lonestar.clinanes
Anesthesia by Dr. Swati Singh	Reference texts/journals, training and education	Pre-operative<br>General anesthesia<br>Surgical specialty<br>Regional anesthesia	Study tool for anesthetics topics	Free	https://play.google.com/store/apps/details?id=com.prepladder.anesthesia
Morgan & Mikhail's Clinical Anaesthesiology Flashcards	Reference texts/journals, training and education	General anesthesia<br>Surgical specialty<br>Regional anesthesia	Flashcard based learning focusing on anesthetics core knowledge	Free<br><br>In-app purchase $44.99	https://apps.apple.com/us/app/morgan-mikhails-clinical-anesthesiology-flashcards/id916009564

App name	App type	Clinical Area	Description	Cost	URL
**Communication**					
AIRS mobile app	Communication	General anesthesia	Online patient safety reporting system	Free	https://qualityportal.aqihq.org/AIRSMain/AIRSCaseEntry

**Electronic Medical Record**					
Powerchart Touch	Electronic medical record	General anesthesia	Mobile access to patient notes, pathology results, clinical photography for users within Cerner network	Free (with eligible Cerner systems)	https://www.cerner.com/solutions/powerchart-touch

TIVA: Total intravenous anesthesia.

Supplementary Figure S1 Worked Example

"I am a predominantly adult anaesthetist but work in a rural/ regional centre and must occasionally anaesthetise children. I am not the most technologically literate but use an iPhone."

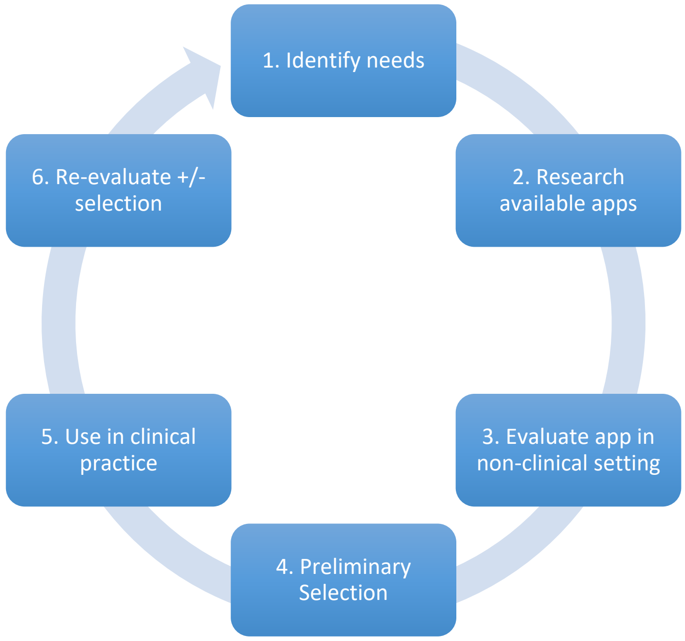

1. *Identify needs*

   I have considered aspects of my practice that would be augmented by technology, and have decided Paediatric dose calculations are something I frequently double check, and would be improved with an app.

2. *Research available apps*

   I have discussed with paediatric anaesthetic colleagues, registrars and collated a list of potential apps, as well as used the Google Search engine and Apple App store to identify further options.

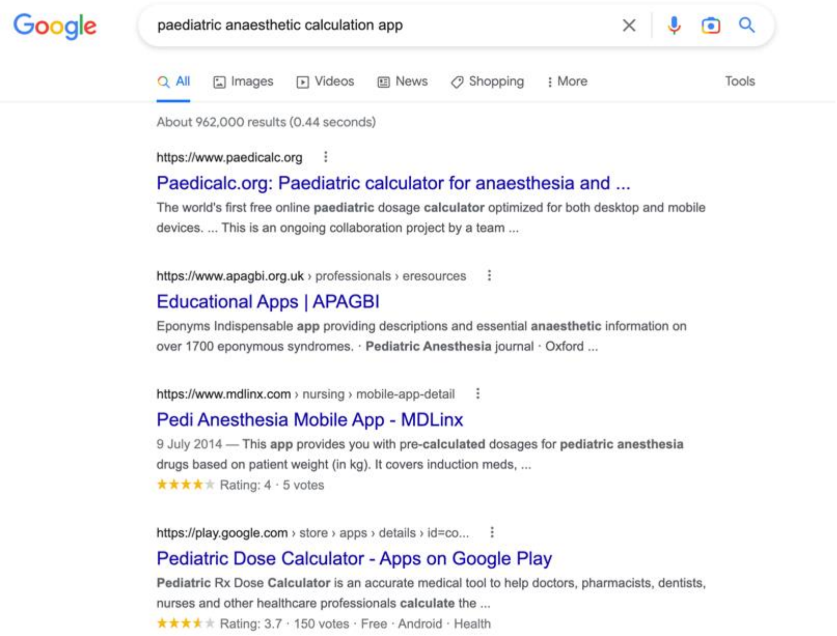

Additionally, I have looked up the most promising apps in journals and websites such as iMedical Apps, and looked at reviews.

3. *Trial app in a non-clinical setting*

From the searches and reviews above, I have narrowed the choice down to around 3 potential apps, and downloaded these onto my phone. Using the provided Table 1, it looks as though app functionality and cost will be my two main criteria. Evaluating the provided apps using Table 1, there is one app I think will be most suited to my needs.

4. *Preliminary selection*

After conferring with colleagues and reading reviews more carefully for the selected app, I agree that this app is one I will trial.

5. *Use in clinical practice*

I will use the app in theatre, noting the aspects of my work it improves, and areas where the app perhaps falls short.

6. *Re-evaluate the app*

After a period (I will select a month), I will take out some conscious time and evaluate the app, and evaluate using the rubric in Table 1 whether I will continue to use the app.
